# DermatoFibroSarcoma Protuberans: A case report of a complete cure

**DOI:** 10.1002/ccr3.6218

**Published:** 2022-08-10

**Authors:** Prajwal Pudasaini, Sushil Paudel, Sadiksha Adhikari, Monique Kafle

**Affiliations:** ^1^ Civil Service Hospital Kathmandu Nepal; ^2^ Patan Academy of Health Sciences Lalitpur Nepal

**Keywords:** DermatoFibroSarcoma Protuberans, storiform collagenoma

## Abstract

DermatoFibroSarcoma Protuberans (DFSP) is a rare recurrent fibrohistiocytic tumor. Given the limitation of available diagnostic modalities in a resource poor setting, diagnosis can be confusing. As most of the tumors recur with time, our case of complete cure was interesting phenomenon observed in our case.

## Graphical abstract

DermatoFibroSarcoma Protuberans (DFSP) is a rare recurrent fibrohistiocytic tumor. Because of the rarity of the disease, non‐specific presentation and lack of immunohistochemistry (IHC), its diagnosis is often delayed and prompt treatment is deferred, which can lead to complications such as recurrence and metastasis. Given the limitation of available diagnostic modalities in a resource‐poor setting, diagnosis can be confusing. As most of the tumors recur with time, our case of complete cure was an interesting phenomenon observed in our case.

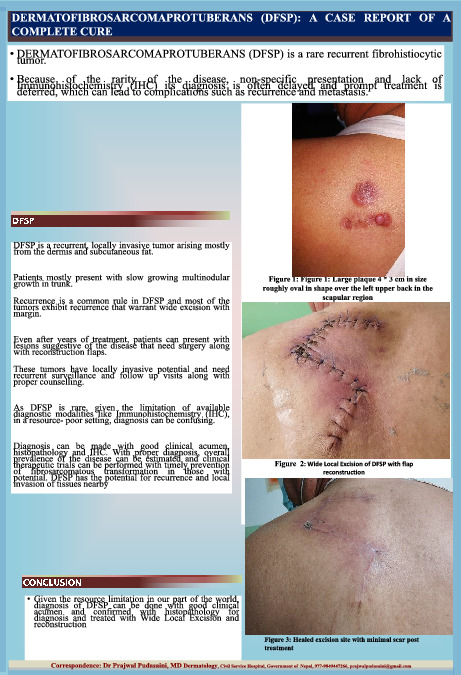

## 1 BACKGROUND

DermatoFibroSarcoma Protuberans (DFSP) is a rare, slow growing, recurrent tumor arising mostly from the dermis and subcutaneous fat. Patients present with slow growing multinodular solitary growth in trunk with recurrence post treatment.^1^ DFSP is a CD34‐positive tumor with a predominance of spindle cells in the histopathological sections. The etiological association with the COL1A1‐PDGFB has led to the dramatic response to the new therapeutic lineage of tyrosine kinase inhibitor–imatinib mesylate.^2^ Given the lack of availability of the FDA‐approved targeted therapy, imatinib mesylate for recurrent DFSP in a resource‐poor setting like ours, priority should be given toward wide local excision with 2–4 cm margin in its treatment. The median age of onset of disease is around 40 years with male predominance.^3^ The histopathology of DFSP, which is the confirmatory test, shows storiform collagenoma with pleomorphism of the spindle cells of the dermis.^4^ The mitotic rate in the spindle cells co‐relates with the metastasis, which is rare.^5^ DFSP is difficult for the clinicians to diagnose and treat because of the rarity of the disease, non‐specific presentation and high degree of recurrence. Here, we report a rare case report of DFSP with complete cure post wide local excision.

## 2 OBSERVATION

**FIGURE 1:**
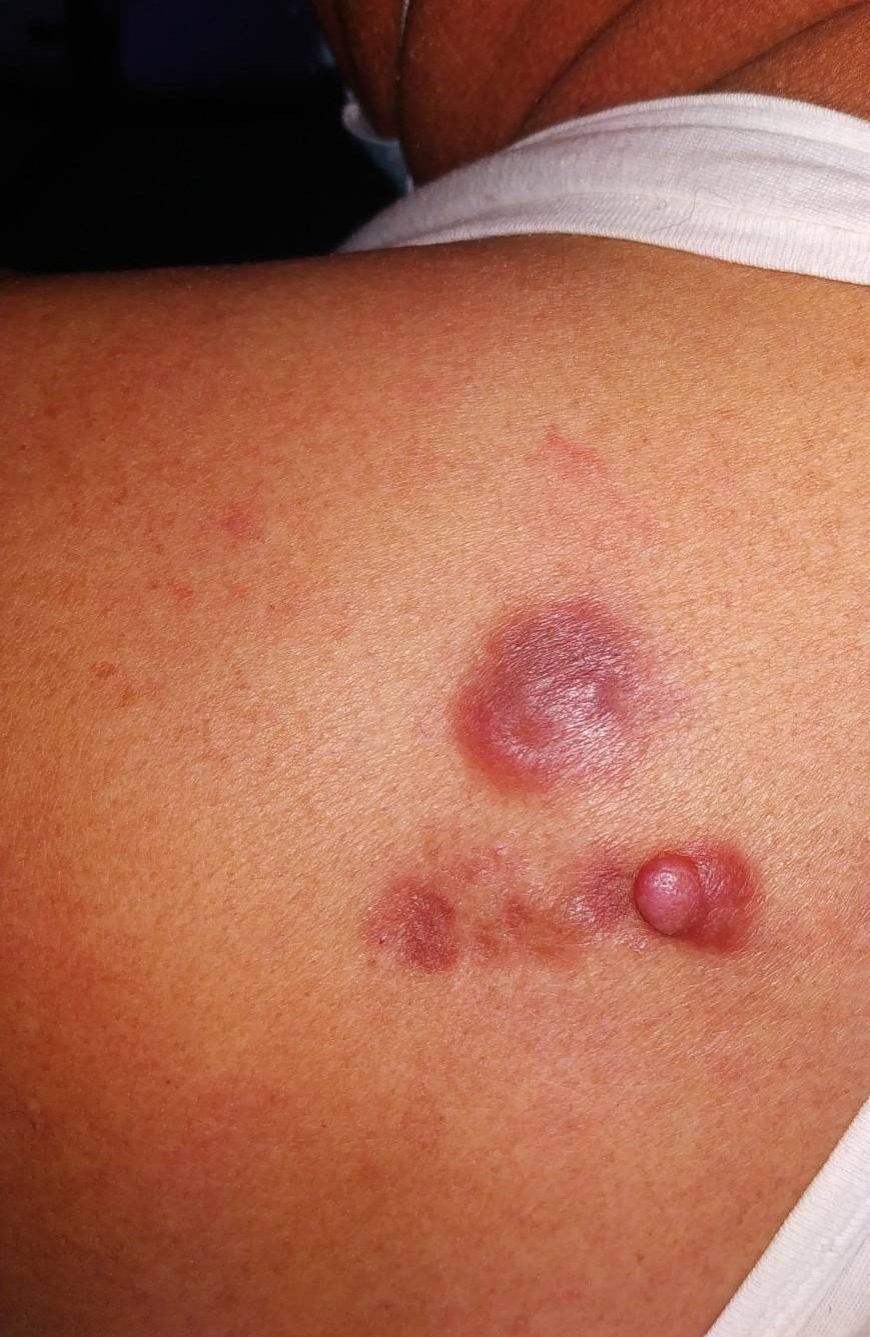
Large plaque 4 × 3 cm in size roughly oval in shape over the left upper back in the scapular region

**FIGURE 2:**
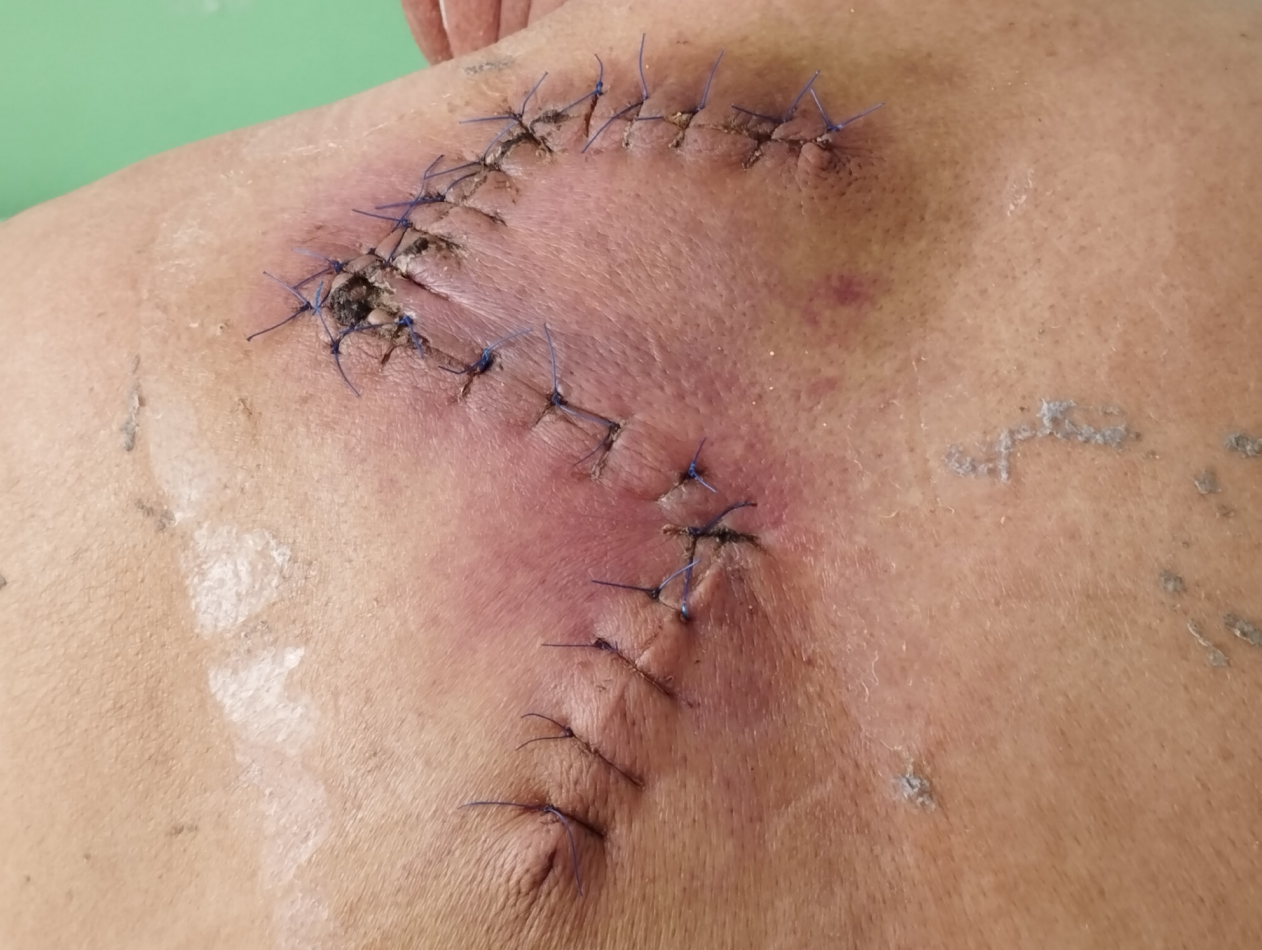
Wide local excision of DermatoFibroSarcoma Protuberans (DFSP) with flap reconstruction

**FIGURE 3:**
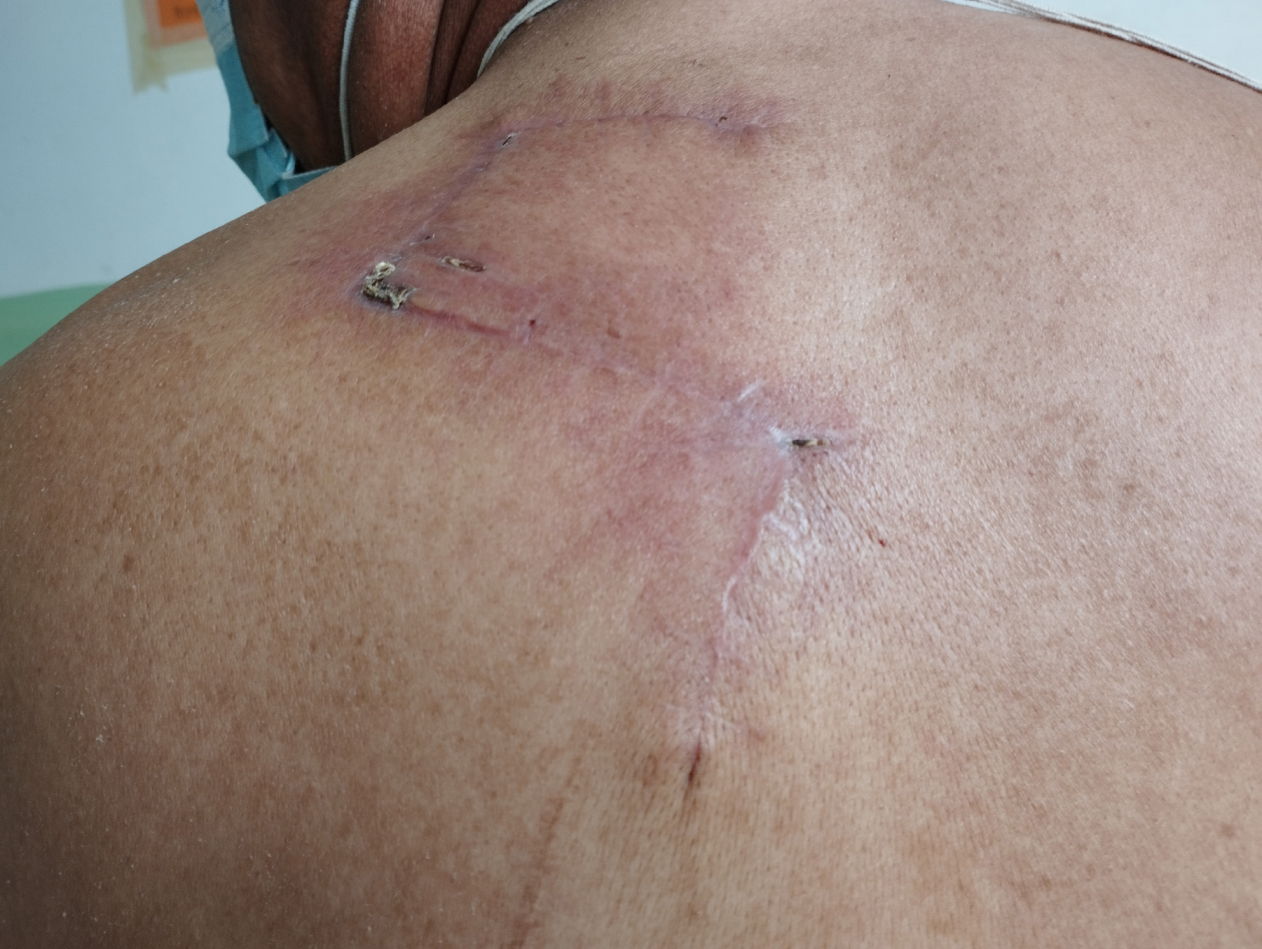
Healed excision site with minimal scar post treatment

A 64‐year‐old male patient from Kathmandu retired serviceman presented with asymptomatic to occasionally painful slow growing lesion over upper back, left scapular region for 16 years. Initially, single pinhead sized, firm, raised lesion in red color was noted over left upper back that increased in size and number with largest one showing multinodular appearance and firm consistency. Lesions evolved over a period of years with 3 in number and larger plaque progressed to form indurated infiltrated plaque with surrounding redness and prominent overlying solitary papule in the other small plaque. On examination, 3 plaques were noted with the largest plaque 4 × 3 cm in size roughly oval in shape over the left upper back in the scapular region, approximately 5 cm from mid‐vertebral line of the spine (Figure 1). Biopsy was done which showed storiform collagenoma of the spindle cells with a whorled pattern. Wide local excision with 2–4 cm margin was done along with reconstruction with Z‐plasty flaps (Figure 2). The excision site with reconstruction healed over time with minimal scarring (Figure 3). There was complete cure of the disease post treatment with no recurrence till date. New lesions have not evolved over the same site for 2 years now, and the lesion has healed with no symptoms and minimal scarring. There was minimal defect post resection with only 5 × 1 cm linear scar achieved with advancement flap.

## 3 DISCUSSION

DermatoFibroSarcoma Protuberans is a recurrent, locally invasive tumor arising mostly from the dermis and subcutaneous fat. Patients mostly present with slow growing multinodular growth in trunk.^1^ It is predominantly seen in in the age range 20–40 years, in which skin‐colored, indurated, multinodular solitary plaque is seen over trunk, shoulder and pelvic areas. There is no gender predilection of the disease.^6^ Recurrence is a common rule in DFSP, and most of the tumors exhibit recurrence that warrant wide excision with margin. Even after years of treatment, patients can present with lesions suggestive of the disease that need surgery along with reconstruction flaps.^7^ These tumors have locally invasive potential and need recurrent surveillance and follow‐up visits along with proper counseling.^8^ As DFSP is rare, given the limitation of available diagnostic modalities such as immunohistochemistry (IHC), in a resource‐poor setting, diagnosis can be confusing. Diagnosis can be made with good clinical acumen, histopathology and IHC. With proper diagnosis, overall prevalence of the disease can be estimated and clinical therapeutic trials can be performed with timely prevention of fibrosarcomatous transformation in those with potential. DFSP has the potential for recurrence and local invasion of tissues nearby.

## AUTHOR CONTRIBUTIONS

PP and SP contributed to the collection of data and the management of the patient. PP and SP wrote the initial draft of manuscript. PP, SP, SA, and MK revised and prepared the final version of the manuscript. All authors have read and approved the final manuscript and agreed to take full responsibility for the integrity and accuracy of the work.

## CONFLICT OF INTEREST

None.

## CONSENT

Written informed patient consent has been signed and collected in accordance with the journal's patient consent policy.

## Data Availability

The data that support the findings of this study are openly available in Clinical Case Reports.
